# Relationship between perceived material living conditions and subjective health and wellbeing as moderated by personal attributes in a representative sample of Nigerians

**DOI:** 10.3389/fpsyg.2025.1530302

**Published:** 2025-05-12

**Authors:** Dare A. Fagbenro, Erhabor S. Idemudia, Klaus Boehnke

**Affiliations:** ^1^TWAS-DFG Research Fellow, Constructor University Bremen, Bremen, Germany; ^2^Department of Psychology, University of Ilorin, Ilorin, Nigeria; ^3^Faculty of Humanities, North-West Potchefstroom, Mafikeng, South Africa; ^4^Bremen International Graduate School of Social Sciences (BIGSSS), Constructor University Bremen, Bremen, Germany

**Keywords:** subjective health, wellbeing, perceived material living conditions, personal attributes, Nigerians

## Abstract

**Background:**

Previous research has linked material living conditions to subjective health and wellbeing. However, moderators are mainly unknown. Thus, the current study examined whether gender, age, and education moderate Nigerians’ subjective health and wellbeing, considering their material living conditions.

**Methods:**

The 2023 Afrobarometer survey in Nigeria’s six geopolitical zones included 1,600 adults aged 18–97 (Mean age = 34.93 years, standard deviation = 13.12, female = 51.9%). IBM SPSS Amos 23 performed SEM and multi-group analyses.

**Results:**

The study found that insufficient material living conditions harm subjective health and wellbeing. It revealed that such conditions harmed subjective health and wellbeing, regardless of age or gender. Surprisingly, education affected the relationship between material living conditions and subjective health and wellbeing, particularly among highly educated individuals.

**Conclusion:**

The study concluded that poor living conditions harm health and wellbeing, whereas education moderates the relationship between material living conditions and subjective health and wellbeing. These findings highlight the need for psychological interventions and policies to improve Africans’ health and wellbeing.

## 1 Introduction

Over the years, the subjective health of individuals has been a critical focus of global research. This is due to the positive outcomes associated with the concept; for instance, subjective health is positively linked with longevity, better body functioning, and a more positive body image (Diener and Chan, 2011; Martín-María et al., 2017; Sollerhed et al., 2021). Previous research has established that material living conditions are a vital social factor of health status (Gu and Ming, 2021; Rahkonen et al., 1997). The living conditions, which include access to good roads, electricity, shelter, clothing, safety, and clean water, are essential amenities that citizens should enjoy in any functioning society. Health challenges, such as infectious diseases, nutritional deficiencies, and mental disorders, are often caused by poor living conditions (Myhrvold and Småstuen, 2017). Developed countries like Germany, the United States, and Canada typically provide their citizens with basic living conditions. In a developing African country like Nigeria, however, all these living amenities are rarely present (Orjiakor et al., 2023). The current inflation rate, poverty level, high insecurity occasioned by the fuel subsidy removal, and the devaluation of the Nigerian currency have worsened the provision of basic amenities, which in turn has contributed negatively to the health and wellbeing status of Nigerians (Orjiakor et al., 2023; Raifu and Afolabi, 2024).

**FIGURE 1:**
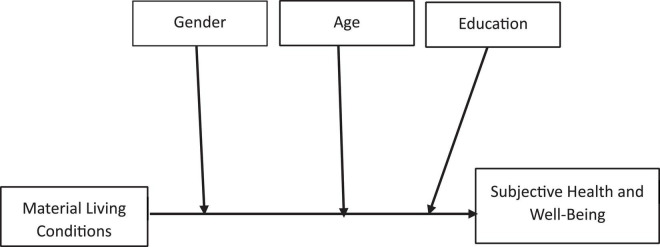
Conceptual model of the study variables.

The present study investigates the relationship between material living conditions and subjective health and wellbeing, and how personal attributes affect this association. Although studies (Ettema and Schekkerman, 2016; Ma et al., 2018) have linked material living conditions to subjective health and wellbeing, the effect of personal attributes on the association between material living conditions and subjective health and wellbeing remains relatively sparse in the literature. However, most studies (Gu and Ming, 2021; Weckroth et al., 2022) on material living conditions and subjective health and wellbeing have primarily been conducted in Western cultures. African settings, specifically Nigerian samples, are largely missing from the literature. This knowledge gap is addressed by the present study, which aims to understand the link between material living conditions and subjective health and wellbeing and to offer insights into the conditions or circumstances that personal attributes (age, gender, and education) may have on this association. The study assumes that favorable living conditions may improve subjective health and wellbeing. This proposition may be influenced by personal attributes such as gender, age group, educational status, and other factors. It is essential to understand the complex interactions between material living conditions and personal attributes on subjective health and wellbeing in an African context, where persistent issues of poor health and wellbeing have been observed among the citizenry (Okechukwu et al., 2022; Orjiakor et al., 2023). Therefore, this study aims to examine the relationship between material living conditions and subjective health and wellbeing and investigate the circumstances under which this relationship may hold, using personal attributes (age, gender, and education) as moderators. The central research questions are: (1) What is the relationship between material living conditions and the subjective health and wellbeing of Nigerians? (2) How do personal attributes (age, gender, and education) moderate the relationship between material living conditions and the subjective health and wellbeing of Nigerians? The Nigeria-focused study thus proposes the following hypotheses: (H1) The more negatively living conditions are evaluated, the lower the subjective health and wellbeing among Nigerians; (H2) The relationship between the perceived quality of material living conditions and subjective health and wellbeing will be stronger among women; (H3) The younger Nigerians age, the stronger the relationship between perceived material living conditions and subjective health and wellbeing; (H4) The more highly educated respondents are the higher the correlation between the perceived quality of their living conditions and subjective health and wellbeing. The research conceptual framework shows the possible interplay between the perceived material living conditions and personal attributes in influencing subjective health and wellbeing, as illustrated in Figure 1. Through this investigation, the study aimed to strengthen and enrich the existing literature on material living conditions, personality attributes, and subjective health from a theoretical perspective. Exploring these interactions is also vital for providing psychological interventions as a guide for policymakers to formulate strategies to enhance Africans’ subjective health and wellbeing, specifically Nigerians.

## 2 Literature review

### 2.1 Material living conditions and subjective health and wellbeing

Living conditions can negatively or positively impact physical and mental health, and good living conditions can help improve individual mental health (Barskova and Oesterreich, 2009; Evans et al., 2000). The World Health Organisation and the World Bank have called for improved self-rated health (SRH) among citizens through improved living conditions (WHO, 2015). Research on living conditions and subjective health has been well-established in the literature; for instance, Gu and Ming (2021) found that individuals living in high-quality houses reported better health more frequently than those living in ordinary homes. Olukolajo et al. (2013) found that poor housing conditions have a detrimental impact on human health. As such, the presence or absence of good living conditions may predict an individual’s subjective health. However, to what degree is that the case in an African context? Most research comes from the Global North. There is a lack of research exploring the direct link between material living conditions and subjective health in the Global South among Nigerians.

The literature on subjective wellbeing (SWB) generally views good subjective health as a crucial element of SWB (Diener, 2025; Dyar et al., 2022). According to Moore and Diener (2019) and Liebenberg and dos Santos (2018), subjective wellbeing, which is defined as life satisfaction with positive and negative effects, refers to ability to delve into life with positivity and have positive, enjoyable, and exciting experiences without suffering from distress, fear, or anxiety. Additionally, SWB is the degree to which a person thinks their life is going well (Diener et al., 2018). SWB can be situated on a continuum, ranging from very low to very high wellbeing states (Abdel-Khalek and Lester, 2013). Research on the link between material living conditions and subjective wellbeing (SWB) has recently gained attention among academics and policymakers (Ettema and Schekkerman, 2016; Weckroth et al., 2022). Growing evidence from the literature suggests that SWB and indicators of living conditions are indeed closely related. According to Aretz et al. (2019) and Levinson (2020), contextual factors, such as issues with public transportation, negatively impact wellbeing. Additionally, Rüger et al. (2023) found that the quality of housing and public goods is strongly associated with subjective wellbeing (SWB). Further studies (e.g., Ettema and Schekkerman, 2016; Ma et al., 2018; Weckroth et al., 2022) have found a strong link between the immediate living environment and physical health. Likewise, Ma et al. (2018), Balducci and Checchi (2009), and Dittmann and Goebel (2010) all found that crime, existential insecurity, and general Neighborhood problems have a negative impact both on mental health and SWB. Scholars such as Ettema and Schekkerman (2016) or Weckroth et al. (2022) stress the need for more investigation into the direct link between material living conditions and wellbeing, especially in developing countries. This study responds to this call by investigating the impact of material living conditions on subjective wellbeing in an African setting, highlighting the necessity for further research in this area.

The association between material living conditions and subjective health and wellbeing is captured in the bottom-up theory of SWB (Diener, 1984; Marans and Rodgers, 1975), which proposes that diverse factors in a life domain, such as living environment conditions, either unfavorable or favorable, determine an individual’s overall subjective health and SWB. Hence, a range of adverse living conditions, such as poor electricity, inadequate road networks, and unstable power supply, that people perceive can negatively predict subjective wellbeing and life satisfaction among the Nigerian population. This nexus has often been neglected in wealthy countries of the Global North.

Readers may wonder whether such subjective measures are not overly biased. In line with the classical Thomas Theorem (Thomas and Thomas, 1928), it is clear that only material living conditions that individuals perceive can impact people’s behavior. In other words, not the rain that fell, but the rain perceived as having fallen is essential.

### 2.2 Personal attributes as moderators

Not all individuals who experience adverse material living conditions will report low levels of health and wellbeing. DeNeve and Cooper (1998) and Lucas (2018) asserted that the living conditions of people do not influence SWB, but rather their specific personal attributes that can underlie this association. Numerous researchers have agreed that individual characteristics are indeed related directly or indirectly to subjective wellbeing (Abdullahi et al., 2019; Easterlin, 2003; Easterlin et al., 2010; Helliwell et al., 2012; Myers and Diener, 1995), but the causal link has continued to be a subject of debate (Clark et al., 2008; Lyubomirsky et al., 2005). This present study considers specific personal attributes (such as gender, age, and education) that may explain the conditions or circumstances that may influence this association. These three personal attributes are further elaborated upon concerning material living conditions and subjective health and wellbeing.

#### 2.2.1 Gender

Gender is one personal attribute that can be defined as a person’s status as either male or female (Ogungbamila and Olaseni, 2019). According to Ojedokun (2015) and Ogungbamila and Fajemirokun (2016), the concept of gender role refers to the sociocultural and physiological roles that are typically associated with males and females in a given social setting (Boehnke, 2011). Subjective health and wellbeing can be perceived differently depending on one’s gender. Theoretically, the gender intensification hypothesis (Avison and Mcalpine, 1992) also proposed that males and females experience life pressure because of what is culturally acceptable for each gender, and their ability to confront these pressures is a result of their social or biological differences, which are linked to their wellbeing (Chen et al., 2013; Ge et al., 2001; Overholser et al., 1995). There have been recent and abundant studies on the relationship between gender and subjective wellbeing; for instance, Fumagalli and Fumagalli (2022) found that men and women differ in their levels of subjective wellbeing. Kurnia et al. (2021) found that gender significantly impacts subjective wellbeing (SWB). In their study, Esteban-Gonzalo et al. (2020) investigated gender differences in subjective wellbeing indicators among 1,407 children and adolescents in Spain. Their result revealed significant differences, with women having higher subjective wellbeing than their male counterparts. More recently, Buhner et al. (2022) investigated gender differences in subjective wellbeing among 75 married couples from Altai Krai. It was found that there were gender differences in subjective wellbeing among the sampled respondents. Abdullahi et al. (2019) investigate gender as one factor that affects SWB among 732 participants from Nigeria. The study found that men focus more on social wellbeing and life satisfaction components, whereas women focus more on emotional wellbeing. Bücker et al. (2018) report that gender is only indirectly associated with subjective wellbeing (SWB), whereas Joshi (2010) found that gender did not significantly influence the SWB of either males or females, implying that males and females similarly evaluate their wellbeing.

#### 2.2.2 Age

Age is another personal factor linked to subjective health and wellbeing (Hou et al., 2022; Ronen et al., 2016). Over the years, numerous scholars have found that subjective wellbeing (SWB) declines with age (Lacey et al., 2006), whereas others argue that wellbeing remains stable or increases in later life (Wettstein et al., 2016). Subjective wellbeing declines with age, which may be particularly true for older individuals in Nigeria, where support for older age is limited (Mbam et al., 2022), and their conditions are often more dependent (Olawa, 2024). Younger age groups are expected to be more agile and rely more on friends for support in the event of any life stressor, which may indirectly impact their subjective health and wellbeing. The relationship between age and subjective health and wellbeing is supported by the hedonic treadmill theory (Kahneman, 1999), which suggests that as people age, they experience various life changes, including shifts in health, social roles, and life circumstances, which may lead to a decline in their wellbeing. The literature remains conflicted regarding the relationship between age and subjective health and wellbeing. For instance, Biermann et al. (2022) found no evidence of a U-shaped association between subjective wellbeing and age. Ronen et al. (2016) found that emerging adults (aged 19-24 years) exhibit lower levels of subjective wellbeing (SWB) compared to adolescents aged 12-18 years. Abdullahi et al. (2019) found in Nigeria that emerging adults (under 24 years old) were associated with better social wellbeing (SWB) and happiness than older adults aged 24 years and above. Hou et al. (2022) found that age indirectly moderates the link between education and subjective wellbeing. Additionally, Kassenboehmer and Haisken-DeNew (2012) found that age was not associated with subjective wellbeing. Furthermore, Agrawal et al. (2011) reported a statistically significant relationship between age and specific components of SWB. Deaton (2007) found that in Eastern European and former Soviet Union nations, life satisfaction declined with age. According to research by Bălţătescu (2014) and Della Giusta et al. (2011), who studied Romanians and British citizens, respectively, life satisfaction among these populations decreases with age.

#### 2.2.3 Education

Education in this context refers to individuals with no formal schooling and individuals with some formal schooling. Formal learning is often acquired over a specific period and may be attained from high school to tertiary institutions. Education attainment has also been established to have a strong link with subjective health and wellbeing, but the relationship also needs further investigation (Shervin and Bazargan, 2019; Wang and Sohail, 2022). Education exerts both direct and indirect effects. The direct effect encompasses benefits for a well-educated individual, including increased self-confidence, higher self-esteem, and enhanced health-related self-care. In contrast, the indirect effect helps improve job opportunities, employment, and socioeconomic status, which may lead to better quality treatment and enhanced health and wellbeing. According to the Minorities’ Diminished Returns (MDRs) theory (Assari and Bazargan, 2019; Shervin and Ritesh, 2019), specific health disparities are attributed to the less protective effects of educational attainment on the health and wellbeing of some individuals in society than initially anticipated. Hence, educated individuals may find ways to cope effectively with their living conditions, thereby experiencing better health and wellbeing. In contrast, individuals with limited education may face a diminished ability to cope with their living conditions, which can negatively impact their health and wellbeing.

The relationship between education and subjective wellbeing has been examined in numerous studies (Arpino et al., 2018; Yakovlev and Leguizamon, 2012). In their research, Wang and Sohail (2022) discovered a strong correlation between education and an improvement in one’s subjective wellbeing. Hou et al. (2022) found that education has a significant positive relationship with the subjective wellbeing (SWB) of rural dwellers in China. Additionally,

Ndayambaje et al. (2020) found that individuals with higher education tend to be generally happier and more content than those with lower education levels. Furthermore, Jongbloed (2018) asserts that education enhances society’s living conditions and improves overall wellbeing. A study by Clark and Lepinteur (2019) established that unemployed individuals with higher levels of education were less content than those with lower levels of education. Additionally, Assari and Bazargan (2019) found that better subjective health and wellbeing were associated with higher levels of educational attainment. In another study, Helliwell et al. (2012) found that education significantly impacts subjective wellbeing. Most studies indicate a consistent trend: higher education is associated with better subjective wellbeing (SWB). However, the picture is straightforward in its uniformity. More highly educated individuals, for example, tend to worry more about distal phenomena, such as wars and environmental destruction, which may harm their subjective wellbeing (Smallenbroek et al., 2023).

## 3 Materials and methods

### 3.1 Study design and sample

The study utilized freely accessible data from the 2023 Afrobarometer,^1^ which surveyed 1,600 Nigerian adults between March 5 and 31, 2023, across all 36 states in Nigeria, including the Federal Capital Territory (FCT). The 36 states and the FCT are subdivided into six geopolitical zones: North East, North Central, North West, South East, South West, and South South.

The Afrobarometer is an African, non-partisan research network that surveys public opinion on governance, democracy, wellbeing, economic conditions, and other related issues in over 42 African countries, including Nigeria. As a population-based survey of participants, the Afrobarometer consists of large cross-sectional and some longitudinal samples drawn using national probability sampling, thereby securing a nationally representative, randomly stratified database. The survey network often employs the stratified random sampling method, which involves dividing populations into various strata (such as regions, urban versus rural areas, etc.) to ensure that all relevant groups are represented. This stratification ensures that the sample is diverse and reflects Nigeria’s demographic composition. For the sample size, the Afrobarometer survey typically targets a 95% confidence level; hence, it often uses a desired margin of error of ± 2–3%. This smaller margin of error requires a larger sample size. This is why a sample size of *N* = 1,600 was used for the survey. Afrobarometer survey data have been well utilized by scholars in academic papers and doctoral theses (Isbell, 2022; Diallo, 2022; Alabi and Olajide, 2023). Sampling was done using multi-step random selection methods and proportionate probability for persons aged 18 and above in Nigeria. Gender-balanced interviewers conducted all interviews face-to-face, with men interviewing male respondents and women interviewing female respondents. For more details on sampling procedures, please visit the Afrobarometer website at https://www.afrobarometer.org/surveys-and-methods/sampling.

### 3.2 Measures

All variables in the study were adopted from the 2023 Afrobarometer data set. Due to the partial reliance on self-report measures, self-report biases, including social desirability and response set biases, have a relatively high potential. These biases may skew the findings in a particular direction or increase errors in the study.

#### 3.2.1 Outcome variables

Five items were utilized to assess respondents’ subjective health and wellbeing. The items read: “Over the past year, how often, if ever, have you or anyone in your family gone without enough food to eat?” “… enough clean water for home use?” “…medicines or medical treatment?” “… enough fuel to cook your food?” “… a cash income?” Respondents were asked to rate the items on a 5-point scale, ranging from 0 (never) to 4 (always). Missing responses were coded as –1, and “don’t know” was coded as 9. The consistency coefficient α of the 5-item index was 0.90; *note that high scores indicate low subjective wellbeing*.

#### 3.2.2 Predictor variables

Material living conditions were assessed by asking the *interviewer* to rate the following five items: “Are the following facilities present in the primary sampling unit or in easy walking distance?” (1) post office, (2) police station, (3) health clinic (private or public or both), (4) social center, government help center, or other government office where people can request help with problems. The fifth item asked the interviewer, “In the primary sampling unit/enumeration area, did you (or any of your colleagues) see (5) any soldiers or army vehicles? Items had to be answered with either 1 = “yes” or 0 = “no,” with –1 (missing) and 9 (for Items 1-4, indicating “can’t determine,” or for Item 5, “don’t know”). A higher score is seen as speaking to better material living conditions. The Kuder-Richardson’s α was 0.93 for the 5-item index.

#### 3.3.3 Moderating variables

The personal attributes (gender, age, and educational attainment) were modeled as moderating variables and were measured using the demographic variables. Gender was measured as male (0) or female (1). Educational attainment was recoded as “no education” (1) vs. “educated” (2). Age in the data set was measured continuously in years, but it was recoded into two groups: 18-49 years as the younger age group (1) and 50 and above as the older age group (2).

### 3.4 Statistical analysis

All analyses were conducted using IBM SPSS Statistics for Windows, Version 27, and IBM AMOS Version 24 software (Armonk, NY, United States; Arbuckle, 2016). Descriptive and inferential statistics were employed to analyze the collected data. Descriptive statistics were calculated for participants’ sociodemographic characteristics, including frequencies, means, and standard deviations. Pearson’s bivariate correlations were used to assess the associations between variables. Structural equation modeling with maximum likelihood estimation was used to test the direct relationship between material living conditions and subjective health and wellbeing. Several different coefficients were used to assess the goodness-of-fit of the SEM model, which includes the goodness-of-fit index (GFI), Tucker-Lewis index (TLI), comparative fit index (CFI), incremental fit index (IFI), root mean square error of approximation (RMSEA), and the standardized root mean square residual (SRMR). The goodness-of-fit is acceptable if the GFI, TLI, CFI, and IFI values are higher than 0.90, and excellent if they are higher than 0.95. Values of RMSEA and SRMR should be lower than 0.08 (Browne and Cudeck, 1992; Hu and Bentler, 1999) for well-fitting models. Furthermore, a multigroup SEM analysis was employed to investigate the moderating effect of personal attributes (gender, age, and education) on the relationship between material living conditions and subjective health and wellbeing. The χ^2^ score was compared between the unconstrained and constrained models, where path coefficients were fixed to equality. If the unconstrained model exhibits a significantly better fit when comparing the χ^2^ scores of both models, it is concluded that there is some form of moderation effect in the structural model. To assume a moderation between two groups, Δχ^2^, the difference between the χ^2^ scores of the unconstrained and constrained models, must be 3.84 or 6.63 to produce a significant difference at *p* < 0.05 and *p* < 0.01, respectively.

## 4 Results

### 4.1 Data screening

The dataset contains 1,600 participants, who were checked for accuracy and completeness. Due to the secondary nature of the dataset, missing data were excluded before uploading the dataset from the Afrobarometer website. A careful observation of the data downloaded by the researchers reveals indeed no missing values in the dataset. The univariate normality test was done using the skewness and kurtosis indices. The analysis showed that the skewness values for the variables ranged from –0.07 to –1.97, while the kurtosis values varied from –1.19 to 3.11. These values are within Byrne (2010) and Kline (2011) suggested range of ± 3 for skewness and only minutely outside these boundaries for kurtosis.

Multicollinearity was checked using the Tolerance Value and Variance Inflator Factor (VIF). The analysis also revealed that tolerance values were between 0.98 and 0.99, which are within the threshold of lower than 0.1, whereas Variance Inflator Factor (VIF) values were between 1.00 and 1.01, also within the range of less than 10 (Hair et al., 2010; Tabachnick and Fidell, 2006).

### 4.2 Respondents’ socio-demographics and Pearson’s correlation results

Descriptive statistics (Table 1) were used to document the respondents’ socio-demographic characteristics. It was observed that the majority, 830 (51.9%), were male, whereas 770 (48.1%) were female. The age distribution revealed that 827 (51.7%) were between the ages of 18 and 32 years, 773 (48.3%) were between the ages of 33 and 97 years, and the median age for the distribution was 32.00 years. Additionally, most participants, 1,128 (70.5%), were educated, while 472 (29.5%) had no education.

**TABLE 1:** Participant characteristics (*N* = 1,600).

Demographic	Frequency	Median age	%
**Gender**			
Male	830		51.9
Female	770		48.1
**Age**			
18-32 years	827		51.7
33-97 years	773	32.00	48.3
**Education**			
No education	472		29.5
Educated	1,128		70.5

Authors’ calculation from Afrobarometer Data, 2023. SD, standard deviation; %, Percentage.

The correlation matrix (Table 2) was produced to ascertain associations among personal attributes, perceived material living conditions, and subjective health and wellbeing. The results showed that age and subjective health and wellbeing scores were correlated at *r* = –0.05, *p* < 0.05. High wellbeing scores indicate challenging conditions, suggesting that subjective health is evaluated minutely more favorably in the older age group. Gender was unrelated to subjective health and wellbeing (*r* = –0.01, *p* > 0.05). For education and subjective wellbeing, the correlation was *r* = –0.11, *p* < 0.05, suggesting that educated study participants rated their subjective wellbeing better than uneducated study participants. The correlation between material living conditions and subjective health and wellbeing was *r* = –0.17, *p* < 0.05, indicating that better living conditions are associated with fewer subjective health and wellbeing issues.

**TABLE 2:** Means, standard deviations, and correlation among variables.

Variables	Mean	SD	1	2	3	4	5
Age	34.93	13.12	1				
Gender	–	–	–0.14^**^	1			
Education	–	–	0.02	–0.10^**^	14		
Material living conditions	26.48	18.72	0.01	0.06	0.13^**^	1	
Subjective health and wellbeing	21.11	6.13	–0.05*	–0.01	–0.11^**^	–0.17^**^	1

*N* = 1,600; SD, standard deviation; Age (in continuous form); Gender (0, male, 1, female); Education (0, no education; 1, educated).

^**^p < 0.01;

*p < 0.05.

### 4.3 Measurement model

**FIGURE 2:**
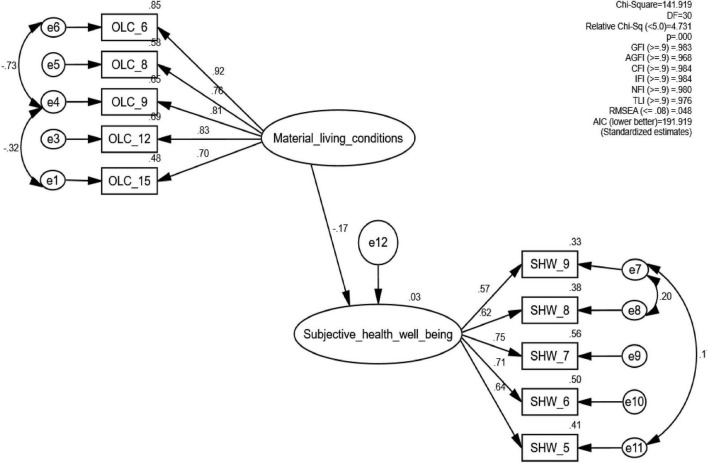
Hypothesized model. OLC, Material Living Conditions; SHW, Subjective Health and wellbeing.

The measurement model ascertains the model fit of the sample data; it is illustrated in Figure 2. The model comprises two latent constructs (material living conditions and subjective health and wellbeing) and ten manifest variables following remodification. An assessment of the fit indices revealed that the measurement model achieved a satisfactory fit: χ^2^(30, *N* = 1,600) = 141.91, *p* < 0.001, CMIN/DF = 4.731, CFI = 0.98, TLI = 0.98, NFI = 0.98, RMSEA = 0.05. All the indicators loaded significantly at *p* < 0.001 on their res*p*ective latent constructs. The measurement model’s construct validity, convergent validity, and discriminant validity were also assessed. It was found that the composite reliabilities for the measures of material living conditions (0.93) and subjective health and wellbeing (0.90) were higher than the recommended cut-off of 0.60 (Bagozzi and Yi, 1988). The convergent validity, assessed through Average Variance Extraction (AVE), was 0.76 for material living conditions and 0.65 for subjective health and wellbeing, both exceeding the cutoff point of 0.50 (Fornell and Larcker, 1981). A check on the two measures also shows that they achieved discriminant validity, as the square roots of the average variance extracted (AVE) were larger than the inter-construct correlations.

### 4.4 Structural model (association between material living conditions and subjective health and wellbeing)

The structural model helps ascertain the direct and indirect effects of the study variables in Figure 2 and Table 3. It revealed that material living conditions negatively predict subjective health and wellbeing (β = –0.16, CR = –5.77, *p* < 0.001). The material living conditions indicate a small effect size (*f*^2^ = 0.03). Considering that the Afrobarometer asks about the frequency of experiencing health hazards in the last 12 months when assessing subjective health and wellbeing, this result aligns with our first hypothesis, H1, that favorable material living conditions are associated with *fewer* health hazards.

**TABLE 3:** Multigroup moderation effect of personal attributes in the relationship between material living conditions and subjective health and wellbeing.

Paths	β	S.E	C.R	*p*	Unconstrained model χ ^2^(df)	Constrained model χ ^2^(df)	Δχ^2^	Decision
Subjective health and wellbeing < – Material living conditions	–0.14 –0.19	0.02 0.02	–3.53 –4.60	<0.001 <0.001	169.03 (60)	169.83 (61)	0.79	Does not moderate
Male								
Female								
Subjective health and wellbeing < – Material living conditions	–0.17 –0.15	0.01 0.04	–5.41 –1.97	<0.001 <0.049	197.55 (60)	198.15 (61)	0.59	Does not moderate
Younger age group								
Older age group								
Subjective health and wellbeing < – Material living conditions	–0.08 -0.20	–0.01 0.02	–1.60 –5.78	0.108 <0.001	183.90 (60)	197.86 (61)	13.96*	Moderate
No education								
Educated								

*p < 0.01. S.E, standard error; CR, critical ratio; *p*, probability level; β, standardized regression weight;

χ^2^(df), chi-square minimum (degree of freedom);

Δχ^2^, Delta chi-squared.

### 4.5 Results of multigroup analysis

#### 4.5.1 Gender differences

The moderation effect on the individual path reveals no apparent difference in the association between material living conditions and subjective health and wellbeing between males (β = –0.14; *p* < 0.05) and females (β = –0.19; *p* < 0.01). The effect size is small (*f*^2^ = 0.03). This result is not in line with our second hy*p*othesis, H2.

#### 4.5.2 Age differences

The individual path analysis reveals that the relationship between material living conditions and subjective health and wellbeing remains consistent across younger and older age groups (β = –0.17, *p* < 0.01 for younger age; β = –0.15, *p* < 0.05 for older age). Age also has a negligible effect size (*f*^2^ = 0.03). This result is not in line with our third hypothesis, H3.

#### 4.5.3 Education differences

The individual path model shows that covariation between objective living conditions and subjective health and wellbeing was found to be significant and relatively strong among educated individuals (β = –0.20, *p* < 0.001) but insignificant among uneducated individuals (β = –0.08, *p* = 0.10). Education differences have a slightly larger, but still essentially negligible effect size (*f*^2^ = 0.04).

This result aligns with our fourth hypothesis, H4.

## 5 Discussion

To the best of our knowledge, this is the first study that investigates the relationship between material living conditions and subjective health and wellbeing, and how personal attributes (gender, age, and education) moderate these relationships among a sample of Nigerians using a multi-group SEM analysis. In line with our first hypothesis, material living conditions were (arithmetically) negatively related to subjective health and wellbeing. This implies that people whose living conditions were assessed by Afrobarometer interviewers as less favorable tend to report lower subjective health and wellbeing. *Note again that the Afrobarometer—unfortunately—used reversed scoring to assess subjective health and wellbeing, so high scores of the variable reflect badly experienced health and wellbeing.* This study’s finding aligns with several studies (Olukolajo et al., 2013; Ma et al., 2018; Balducci and Checchi, 2009; Dittmann and Goebel, 2010), which have found that poor living conditions, characterized by insecurity and general neighborhood problems, harm mental health and subjective wellbeing (SWB).

Corroborating the bottom-up theory of SWB (Diener, 1984; Marans and Rodgers, 1975), Nigerians whose material living conditions are rated as inadequate in terms of, for example, access to postal, health, and social services, do report detrimental subjective health and wellbeing concerning, for example, housing, food, and water supply.

Contrary to our expectations, we found that the relationship between material living conditions and subjective health and wellbeing is not stronger for women; it is the same for both genders. Our findings did not replicate past studies (e.g., Fumagalli and Fumagalli, 2022; Esteban-Gonzalo et al., 2020; Buhner et al., 2022; Abdullahi et al., 2019), which all found gender differences in subjective wellbeing indicators among different respondents in their study. This may be because both men and women in Nigeria often face similar socioeconomic challenges in their daily lives. These differences may be attributed to the evolving nature of equal gender roles and opportunities in Nigerian society, influenced by education, religion, policy changes, and economic factors, which may have contributed to the fact that both males and females do not differ significantly in their health and wellbeing.

Another surprising finding in our study is that age did not moderate the association between material living conditions and subjective health and wellbeing. This implies that both young and older age groups have the same level of association between material living conditions and subjective health and wellbeing. The study finding is in contrast with past studies (Deaton, 2007; Agrawal et al., 2011; Della Giusta et al., 2011; Bălţătescu, 2014; Ronen et al., 2016; Abdullahi et al., 2019; Hou et al., 2022), who all found that age moderated the link between living conditions and their subjective health and wellbeing. This result also did not align with the hedonic treadmill theory (Kahneman, 1999), which suggests that as people age, they experience various life changes. This finding may be connected to the resilient nature of young and older Nigerians and the collectivist culture of sharing and interacting with one another. This may be why there is no difference in their level of health and wellbeing across the two age categories.

The most striking result of our study was that education significantly moderated the association between material living conditions and subjective health and wellbeing; the association was considerably stronger among educated Nigerians. As Dragolov and Boehnke (2025) did in a government-commissioned study in South Africa, we view this as an outflow of entitlement beliefs. Educated Nigerians often perceive themselves as entitled to a better life than their less educated or uneducated counterparts. If then they have to lead their lives under difficult material conditions, they perceive themselves as being treated unjustly and report lower wellbeing than their less educated compatriots. This finding also aligns with Clark and Lepinteur (2019), who found that unemployed individuals with a high level of education were less happy than those with a low level of education. The study finding contradicts previous studies (e.g., Wang and Sohail, 2022; Hou et al., 2022; Ndayambaje et al., 2020; Jongbloed, 2018), who claimed that education raises society’s living conditions and increases its general state of wellbeing. This finding is plausible because getting educated is typically perceived as a means to pursue a better life, and attaining a higher level of education is expected to bring benefits, especially to the average Nigerian. Hence, when the government does not provide these perceived benefits, including conducive living conditions and educated individuals who perceive themselves as having self-worth, they often feel agitated, frustrated, and unhappy. These negative emotions can reduce their health and wellbeing.

### 5.1 Implications of the study

The study variables, material living conditions, and level of education have emerged as essential constructs among Nigerians as they are associated with subjective health and wellbeing. First, evidence has shown that poor material living conditions reduce subjective health and wellbeing. Second, the study helps further understand the moderating role of education as a personal attribute in the negative association between material living conditions and subjective health and wellbeing, with educated Nigerians experiencing this negative association. Thus, the findings of this study have implications for practitioners, specifically psychologists and health experts, to develop support programs where people come together to offer mutual support or infrastructure development interventions aimed at improving the living conditions of Nigerians, which can ultimately enhance the health and wellbeing of Nigerians. At the policy level, our study calls on the government to provide robust and well-coordinated basic social living amenities, including access to affordable housing, uninterrupted power supply, shelter and clothing, clean water, and a sound healthcare system. It is crucial for educated Nigerians to develop a sense that their extra effort to invest in education yields a payoff. Theoretically, the study has strengthened the scientific knowledge base of the existing literature on the relationship between material living conditions, personal attributes, subjective health, and wellbeing. Additionally, the direction of the interaction effect of education as an individual attribute on the relationship between material living conditions and subjective health and wellbeing is novel to this field of study. It can serve as a springboard for future research endevors.

### 5.2 Limitations and future research directions

Despite the valuable insights of the study, there are still some limitations that future research should address. The study employed interviewer data to assess the quality of material living conditions. To address this limitation, future research should incorporate multiple objective data sources, such as peer assessment and behavioral observations. Ideally, future research should involve a longitudinal study to better understand the association between material living conditions, personal attributes, and wellbeing. Furthermore, another limitation is the study’s exclusive reliance on quantitative instruments alone to measure the variables. Due to the complex and multifaceted nature of health and wellbeing, a deeper understanding can be gained through qualitative approaches, such as interviews and focus group discussions, which can provide valuable insights for future researchers. Future research is called to explore whether these findings may be generalized to other African or Global South contexts. These collective research efforts are geared toward developing effective strategies and hope to improve the health and wellbeing of the Nigerian and African population.

## 6 Conclusion

This study offers significant insights into the negative impact of poor material living conditions on declining health and wellbeing in the Global South. The findings emphasize the need for targeted interventions such as support programs and infrastructure development that address poor material living conditions, particularly among educated elites, to enhance their health and wellbeing. This is particularly important because educated elites otherwise will seek their fortune by emigrating to “greener pastures” (Nwana, 2023; Idemudia and Boehnke, 2020) on perilous routes.

## Data Availability

Publicly available datasets were analyzed in this study. This data can be found at: https://www.afrobarometer.org/.

## Footnotes

1
https://www.afrobarometer.org/survey-resource/nigeria-round-9-data-2023
